# Using Pharmacogenomic Testing in Primary Care: Protocol for a Pilot Randomized Controlled Study

**DOI:** 10.2196/13848

**Published:** 2019-08-19

**Authors:** Beatriz Manzor Mitrzyk, Reema Kadri, Karen B Farris, Vicki L Ellingrod, Michael S Klinkman, Mack T Ruffin IV, Melissa A Plegue, Lorraine R Buis

**Affiliations:** 1 College of Pharmacy University of Michigan Ann Arbor, MI United States; 2 Department of Family Medicine University of Michigan Ann Arbor, MI United States; 3 Department of Family and Community Medicine Penn State Health Milton S Hershey Medical Center Hershey, PA United States

**Keywords:** pharmacogenomics, primary care, antidepressive agents

## Abstract

**Background:**

Antidepressants are used by primary care providers to treat a variety of conditions, including (but not limited to) depression and anxiety. A trial-and-error approach is typically used to identify effective therapy, as treatment efficacy and safety can vary based on the response, which is affected by certain gene types. Pharmacokinetic pharmacogenomic (PGx) testing provides phenotypic classification of individuals as poor, intermediate, extensive, and ultrarapid CYP450 metabolizers, providing information for optimal drug selection.

**Objective:**

The objective of this pilot study is to examine the feasibility, acceptability, and preliminary effectiveness of PGx testing when used after starting a new antidepressant medication.

**Methods:**

We are conducting a pilot study with physicians from 6 Department of Family Medicine clinics at the University of Michigan who are willing to use PGx test results to manage antidepressant medication use. From enrolled physicians, patients were recruited to participate in a 6-month randomized, wait-list controlled trial in which patient participants newly prescribed an antidepressant had PGx testing and were randomized equally to have the results released to their primary care physician as soon as results were available or after 3 months. Patients were excluded if they had been taking the antidepressant for more than 4 weeks or if they had undergone PGx testing in the past. Physician participants completed a baseline survey to assess demographics, as well as knowledge, feasibility, and acceptability of PGx testing for this population. At the conclusion of the study, physician participants will complete a survey to assess knowledge, satisfaction, feasibility, acceptability, perceived effectiveness, and barriers to widespread adoption of PGx testing. Patient participants will complete a baseline, 3-month, and 6-month assessment, and control patient participants will have an additional 9-month assessment. Data collected will include the reason for antidepressant use, self-reported medication adherence, side effects, patient health questionnaire 8-item depression scale, generalized anxiety disorder 7-item scale, 12-Item Short-Form Health Survey, work status or changes, and physician and emergency department visits. PGx knowledge and perceptions (including acceptability and feasibility) as well as demographic information will also be obtained.

**Results:**

We recruited 23 physician participants between November 2017 and January 2019, and 52 patient participants between January 2018 and April 2019. Currently, all physician and patient participants have been recruited, and we expect data collection to conclude in January 2020.

**Conclusions:**

This study will examine the preliminary effectiveness of PGx testing after treatment initiation and determine the feasibility and acceptability of PGx testing for use in primary care. Through this study, we expect to demonstrate the benefit of PGx testing and lay the foundation for translating this approach into use within primary care.

**Trial Registration:**

ClinicalTrials.gov NCT03270891; https://clinicaltrials.gov/ct2/show/NCT03270891

**International Registered Report Identifier (IRRID):**

RR1-10.2196/13848

## Introduction

### Background

Antidepressant medications are the third most commonly prescribed drug type in the United States and are taken by 10.7% of American adults [1]. Although antidepressants may be used to treat a variety of conditions, such as insomnia, neuropathic pain, fibromyalgia, migraine, or menopausal symptoms, depression and anxiety are the most common reasons for using an antidepressant medication. In any given 12-month period, up to 20% of US adults visit their primary care physician for anxiety or a depressive episode [2]. More than half of these adults also have a second depressive or anxiety disorder. Almost 60% of individuals treated for depression (58.5%) are prescribed antidepressants by a general practitioner or family doctor, and about 62% of antidepressants are prescribed by general practitioners [3,4].

Antidepressant medication treatment and achievement of a positive clinical outcome are complicated by several factors. It can take 2 to 4 weeks for symptoms to improve [5,6], and it is possible that the antidepressant medication initially prescribed may require dosage adjustments or even a switch to a different medication because of side effects [7]. Alternatively, another antidepressant medication may be added if symptoms are not improved. Thus, the path to effective antidepressant therapy can be long and complicated. Improving the initiation of antidepressant medications, particularly the time course, will improve patient experiences.

Pharmacokinetic pharmacogenomic (PGx) testing of antidepressant drug metabolism by CYP450 genes and the functional variants (CYP2D6, CYP2C9, CYP2C19, and CYP1A2) may potentially assist with determining drug effectiveness and the possibility of side effects for an individual patient and to ensure the medication(s) prescribed are the best choice for that patient. Individuals can be phenotypically classified as poor, intermediate, extensive/normal or ultrarapid CYP450 metabolizers [8]. Theoretically, metabolism is inversely associated with the antidepressant’s clinical effectiveness and side effects. Thus, PGx testing for these genetic variants could be crucial to ensure that antidepressant therapy is tailored to the specific patient. PGx test results should improve the use of antidepressant therapy by minimizing side effects and reducing the number of failed medication trials. These factors in turn should improve medication adherence and antidepressant effectiveness [9]. Although it would be ideal for patients to undergo PGx testing before treatment initiation, waiting for PGx test results, which may take 1 to 2 weeks to return, is not desirable for certain patients, as it would delay treatment and could lead to worsening of their clinical condition. PGx testing early in the treatment process may be beneficial, even if not conducted before treatment initiation. Despite this, the adaptation and implementation of PGx testing into the primary care setting have been slow to occur [10], and PGx testing is not a routine part of primary care practice.

Barriers to adopting PGx testing into clinical practice include issues such as gaps in evidence-based data (insufficient or lacking usefulness), lack of genome-based medicine education, low patient awareness of utility, ethical concerns, inadequate support from prescribers, and inconsistent reimbursement [11]. One key barrier to implementation of PGx testing in primary care is that providers may not be comfortable using PGx data in routine practice, with data suggesting that clinicians commonly override electronic PGx clinical decision support alerts [12]. This suggests a lack of clinician comfort with the integration of PGx data into primary care. In addition, 1 study found that more than 50% of the clinicians surveyed did not expect to use or did not know whether they would use PGx test results in the future [12]. Clinicians may ignore PGx test recommendations if the patient is doing well on the current medication. A possible reason for lack of clinician comfort with PGx testing is limited PGx training [13]. On the basis of this, clinicians may be unprepared to help patients decide if testing is needed, which test(s) to order, or how to interpret and apply test results. Clinician engagement is key to ensuring that PGx test results are effectively adopted into clinical practice and patient care. Data are lacking on the role of PGx testing in primary care to guide antidepressant medication use, and a comprehensive, evidence-based approach is needed.

### Study Objective and Aims

The objective of this pilot study is to examine the feasibility, acceptability, and preliminary effectiveness of PGx testing when available just after, or 3 months after, initiating a new antidepressant medication. In terms of feasibility, we are asking physician participants to self-report barriers to PGx testing, as well as whether they would continue to recommend PGx testing. For acceptability, we are measuring physician and patient participants’ perceptions of PGx testing and whether they think the information is valuable. Finally, the preliminary effectiveness is assessed using antidepressant medication changes, as well as changes in medication adherence, patient symptoms, and health care utilization.

## Methods

### Design

We are conducting a pilot study with primary care physicians and their patients. We enrolled physician participants who agreed to participate in a 1-group pre-post design study, using PGx testing with their enrolled patients. We then recruited patient participants seen by 1 of our participating physicians to enroll in a 6-month, randomized, wait-list controlled trial in which all patient participants newly prescribed (within the last 4 weeks) an antidepressant had PGx testing. The wait-list controlled study design allows us to both have a true control comparison group and also allows us to look at longitudinal effects. Patient participants were randomized equally to 2 groups; one group had their PGx results released to their physician immediately after the baseline visit (intervention), and the other group had results sent to the physician 3 months after their baseline visit (control). All patients enrolled in this trial were recently prescribed one of the target medications by their participating physician. Although PGx test results and any resulting treatment recommendations are made to participating physicians by the study pharmacist, it is up to the physician’s discretion to determine if, and how, test results will be used in clinical care.

The primary outcome for effectiveness is the proportion of patients who were prescribed antidepressant medications which are not contraindicated based on PGx test results. Secondary effectiveness endpoints include the change in symptoms or symptom severity as indicated by changes in the 8-item Patient Health Questionnaire (PHQ-8) depression scale [14] and/or Generalized Anxiety Disorder 7-item (GAD-7) scale scores [15], before and after initiation of an antidepressant, and the change in medication adherence as indicated by the change in Adherence to Refills and Medication Scale (ARMS) scores [16]. Feasibility and acceptability will be assessed by responses to the end of study questionnaires developed for the physicians and participants. This study is approved by the University of Michigan (UM) IRBMED Institutional Review Board (HUM00121185) and registered on ClinicalTrials.gov (NCT03270891).

### Intervention

PGx test results are provided to the clinical pharmacist through the *Informed PGx* assay by Progenity, LLC. This is a PGx assay that interrogates known CYP450 genes among others, which are related to neurotransmitter function and the pharmacokinetics of psychiatric drugs. This assay uses purified genomic DNA, polymerase chain reaction–based amplification of target regions, sample dilution, and sequencing. The following pharmacokinetic genetic variations detected by this assay are used in this study: CYP2D6 *1, *2, *3, *4, *5, *6, *7, *8, *9, *10, *11, *12, *13, *14, *15, *17, *41, and gene duplications; CYP2C9 *1, *2, *3, *4, *5, and *6; CYP2C19 *1, *2, *3, *4, *5, *6, *7, *8, and *17; CYP1A2 *1A, *1C, *1F, *1K, *1L, *3, *4, *5, *6, *7, *8, *11, *15, and *16; CYP2B6 *1, *4, *6, and *18. Polymorphisms in these genes are correlated to rates of metabolism for active or inactive metabolites. On the basis of the presence or absence of polymorphisms, patients are classified as ultrarapid metabolizers, extensive (normal) metabolizers, intermediate metabolizers, poor metabolizers, and unknown metabolizer (unknown). We chose this PGx test because it evaluates variants of CYP450 (CYP2D6 and CYP2C19) that are specific to the primary means of metabolism for certain antidepressant medications. The intervention was designed this way because interpretation of the results and subsequent therapeutic interventions are recommended by Clinical Pharmacogenetics Implementation Consortium (CPIC) guidelines (which are supported by evidence-based medicine) [16]. Progenity, LLC, no longer offers this PGx test commercially but provided it as a research-use assay for this study.

We provided primary care physicians their patients’ PGx test results, interpretation, and antidepressant medication therapy recommendations from our trained clinical pharmacist through a Research Subjective, Objective, Assessment, and Plan (SOAP) note in the electronic medical record (EMR; see Multimedia Appendix 1). The clinical pharmacist uses CPIC guidelines and website recommendations for the target antidepressant medications to support and aid in the interpretation of the PGx test results [17]. The results and interpretation provided are specific to the patient, antidepressant, and any potential interacting coprescribed medications. The delivery of test results through clinical pharmacist replicates routine clinical practice at these sites, as all of our clinics have embedded clinical pharmacists.

### Setting

This pilot study is being conducted with physicians and patients from 6 Department of Family Medicine (DFM) clinics at our academic medical center in 5 neighboring communities.

### Recruitment and Randomization

To conduct this study, we recruited 2 groups of participants (physician and patient participants).

#### Physician Participants

We used convenience sampling to identify physician participants. To be eligible for the study, physician participants (1) must be practicing at 1 of the 6 UM DFM clinics, (2) self-report that they were willing to prescribe antidepressants, (3) must be willing to use PGx test results for the management of antidepressant medications, and (4) must be willing to use PGx test results in the treatment of their patient participants enrolled in the study. Physician participants were recruited through a variety of methods, including presentations at faculty business meetings and clinical site staff meetings, as well as targeted emails or letters and follow-up phone calls. All physician participants completed the consent process and will receive no incentives for their participation in this study.

#### Patient Participants

**Figure 1:**
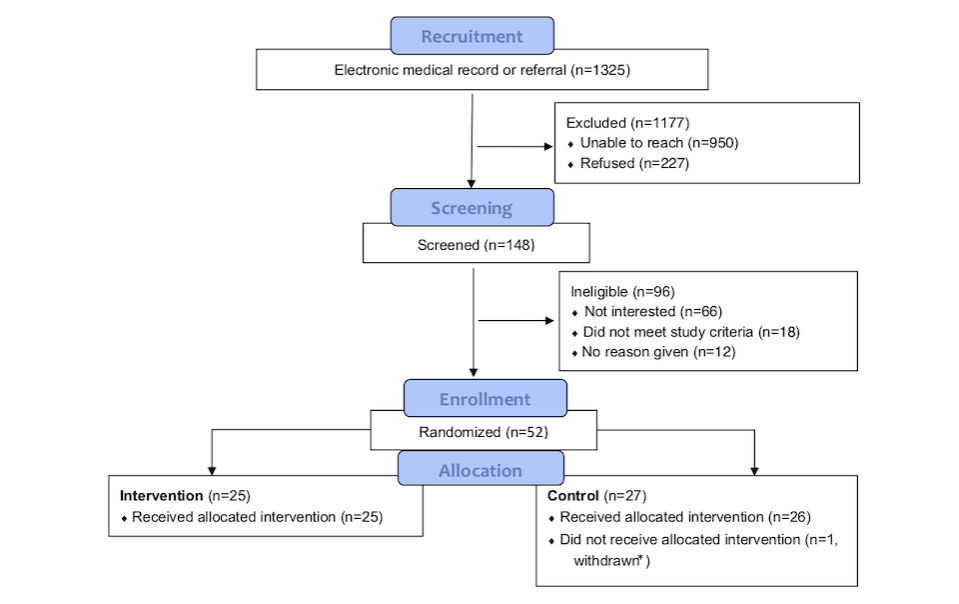
Patient participant flowchart. *Patient was withdrawn from the study 3 months after enrollment because it was discovered that the physician participant listed in the electronic medical record had no previous contact with the patient, and the physician who prescribed the antidepressant did not want to enroll in the study.

Once physician participants were enrolled, we recruited participants from among their patients (Figure 1). To be included in this study, patient participants were required to be (1) a patient of an enrolled physician participant; (2) older than 18 years of age; (3) recently prescribed (with last 4 weeks) and taking one of the following medications: citalopram, escitalopram, fluoxetine, fluvoxamine, paroxetine, sertraline, duloxetine, venlafaxine, nortriptyline, bupropion, mirtazapine, or vortioxetine; and (4) willing to undergo a blood draw for PGx testing. Potential patient participants were excluded if they had previously undergone PGx testing or had been taking the antidepressant medication for more than 4 weeks before enrollment. Patients were also excluded if they were not English speaking or were unable to provide their own consent. Target antidepressant medications for inclusion in this study were selected based on their primary metabolism through CYP2D6 or CYP2C19, or in the case of bupropion, potential for coprescribing with other antidepressants and inhibition of CYP2D6 activity.

We used purposive sampling to identify patient participants. Potential patient participants were identified each week through an automated report from the EMR. Potential participants received 3 targeted recruitment points of contact—initially an email or postal letter, followed by 2 phone calls, emails, or text messages. Patient participants were also recruited through direct provider referral, as well as through posted flyers in the DFM clinics. If a potential participant was eligible to participate in the study, except for the fact that their primary care physician was not yet participating, we attempted to recruit the physician through emails and phone calls.

### Procedures

**Figure 2:**
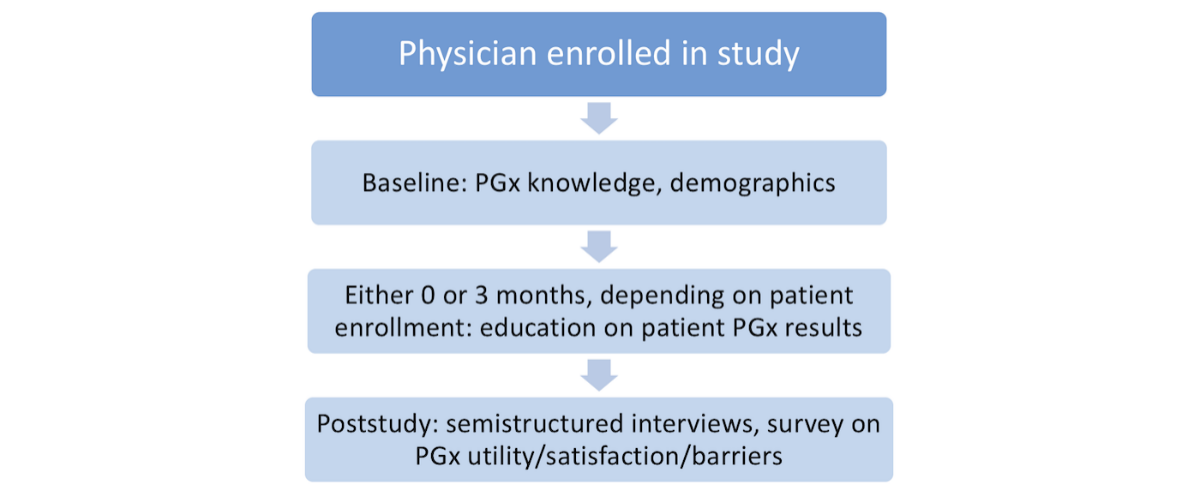
Physician participant study flow. PGx: pharmacogenomic.

Both physician and patient participants were screened for inclusion and exclusion criteria before obtaining consent and enrollment. Once screened as eligible and enrolled, physician participants completed baseline assessments (Figure 2), and we then started to recruit their patients.

**Figure 3:**
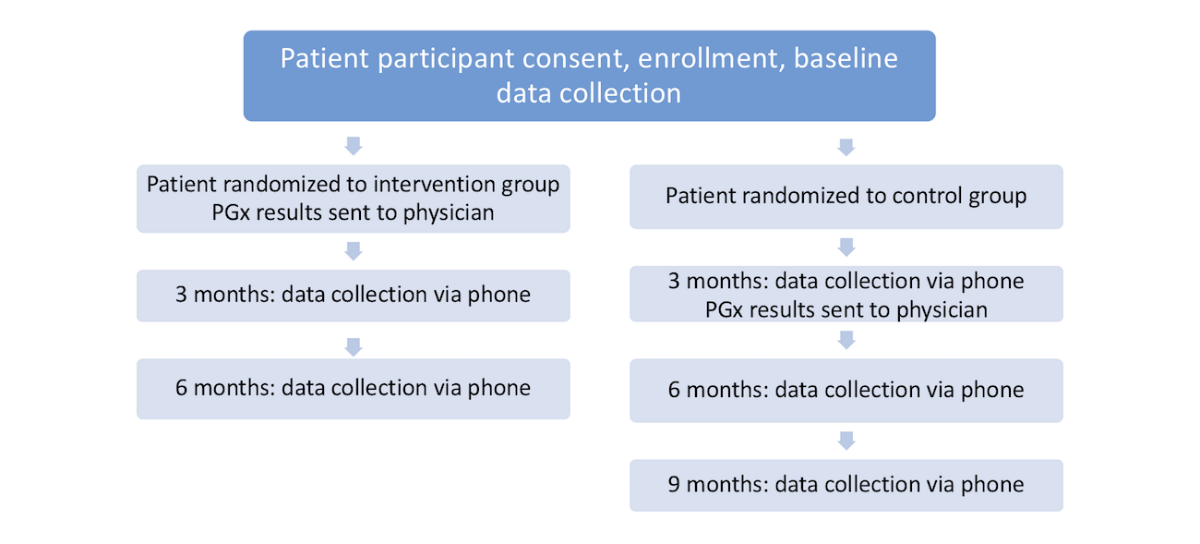
Patient participant study flow. PGx: pharmacogenomic.

Potential patient participants who were screened as eligible gave consent and then completed baseline assessments, as well as had their blood drawn (3 mL) for PGx testing. Patient participants were then randomized to have PGx test results sent to their enrolled physician as soon as the results were available (intervention) or after 3 months (control; Figure 3).

Initially, randomization of patient participants was set to be stratified by physician to ensure balance within provider; however, given the small patient-to-provider enrollment ratio, stratification by provider was not deemed necessary. Instead, we used a block randomization approach, randomizing patients into 1 of the 2 arms using a block size of 6. The randomization schedule was unknown a priori to researchers enrolling patients and revealed to the patient after completion of the baseline survey.

PGx test results were released to physician participants as a SOAP-formatted research note in the EMR for which the physician participant was alerted through electronic notification. The PGx test results and recommendations provided were for the specific antidepressant—enzyme pair (see Multimedia Appendix 1). At a minimum, a trained clinical pharmacist on the research team met for a phone appointment with the participating physician when their first patient PGx test results were available to provide general, as well as patient-specific, education about the PGx test and results. These consultations were intended to help physician participants interpret PGx test results and guide treatment decisions, if necessary. The clinical pharmacist is available to consult with physician participants at any time during the study, and physician participants are encouraged to contact the clinical pharmacist with questions.

No study staff member, including the clinical pharmacist, will provide PGx results to patients; it is up to the physician participants if they choose to share this information with their patients. All intervention patient participants are followed for 6 months, with phone-based follow-up at 3 and 6 months. All control patients are followed for 9 months, with phone-based follow-up at 3, 6, and 9 months.

### Data Collection and Measures

#### Physician Participants

Physician participants completed a self-administered investigator-developed paper survey at baseline. The baseline survey assessed demographics and physician participants’ characteristics, knowledge of PGx testing, and perceptions of the feasibility and acceptability of PGx testing in primary care (Table 1).

At the end of the study, physician participants will self-administer a paper survey. This end of study survey contains similar questions as the baseline survey, with the addition of open-ended questions asking whether physician participants recommended PGx testing to other physicians or whether they ordered PGx testing on patients not enrolled in this study. For the open-ended questions, if the physician agrees and would rather give verbal responses to the questions, their responses will be audio recorded for subsequent qualitative analysis.

Feasibility will be assessed by responses to the poststudy questionnaire, which asks physicians to indicate the extent to which they agree with the statements “I think that the process of learning how to use PGx testing in my daily practice would be easy” and “I believe that I could easily incorporate PGx testing into my clinical practice.” Acceptability will be assessed by responses to the poststudy questionnaire, which asks physicians to indicate the extent to which they agree with the statements “I see the potential benefit of using PGx testing in my clinical practice” and “If I were to recommend that patients undergo PGx testing, I believe that my patients would choose to be tested.”

**Table 1:** Physician participant measures of feasibility, acceptability, and preliminary effectiveness of pharmacogenomic testing within the investigator developed semistructured interview and survey.

Construct	Assessment time	
	Baseline	Poststudy
Demographic information and characteristics of physicians	✓^a^	—^b^
Knowledge of PGx^c^ testing	✓	✓
Acceptability of PGx testing	✓	✓
Utility of PGx testing	✓	✓
Effectiveness of PGx testing	✓	✓
Feasibility of PGx testing	✓	✓
Barriers to PGx testing	✓	✓
Satisfaction with PGx testing	✓	✓

^a^✓ indicates that the construct was assessed.

^b^Not assessed.

^c^PGx: pharmacogenomic.

#### Patient Participants

All patient participants completed a paper baseline survey, had their blood drawn for the PGx test, and had a pill count of all prescription medications completed at baseline. All patient participants are asked to complete the 3- and 6-month follow-up survey and pill count by phone, and control patients have an additional 9-month follow-up survey and pill count. The baseline survey assessed demographics, participants’ characteristics, and health history. All follow-up assessments completed after baseline are telephonic. The baseline and follow-up phone surveys contained questions from the PHQ-8, GAD-7, 12-Item Short Form Survey, Work Productivity and Activity Impairment Questionnaire, the Antidepressant Side-Effect Checklist, and the ARMS. The baseline and follow-up surveys also contained questions from the 3-item self-rated adherence scale, questions about health care utilization, prescribed medications, and ascertained PGx knowledge from an investigator developed survey [14-16,18-20]. The final follow-up survey for each participant includes questions assessing feasibility through responses to statements such as “Did your physician share the PGx test results with you?” and an indication of how much the patient participant agreed with the statement “I understood the PGx test results as explained to me by my doctor.” Moreover, acceptability is assessed in the final follow-up survey by responses to indicate how much the participant agrees with the statements “The PGx test results were valuable” and “I would recommend this PGx test to a friend with my condition.” We collect a complete list of all medications prescribed from the EMR at all assessments, and pill counts are conducted in person at baseline and over the phone at follow-up assessments. Table 2 presents a complete list of patient participant measures and assessments.

### Data Analyses

All survey data, including patient and provider demographics and characteristics, will be analyzed using descriptive statistics to ensure data quality. Independent samples *t* test and chi-square tests will be used to compare patients’ characteristics between intervention and control groups. Feasibility, acceptability, and effectiveness based on physician participants’ responses and feasibility and acceptability based on patient participants’ responses will be analyzed descriptively by assessing the responses to the questionnaire items listed above. Descriptive statistics such as mean and standard deviations, frequencies, and percentages will be used, as appropriate. Open-ended responses will be coded using qualitative thematic text analysis, including any transcripts that result from the recorded survey responses. A team of researchers will analyze and code data independently after initially reading transcripts to gain a general sense of the data, then conduct open coding to identify units of meaning to segments of text, and eventually group codes into major themes and subthemes.

Effectiveness using patient data will be assessed using statistical models. Whether the patient participant is currently prescribed an antidepressant that is appropriate (not contraindicated) based on the PGx test result will be assessed either as soon as results are available or 3 months after the baseline visit, depending on randomization. Logistic regression models, using a generalized estimating equation framework to account for within-subject dependence, will assess the effect of the intervention on the likelihood of being prescribed an appropriate medication by including time, study group, and time by study group interaction as predictors. Secondary effectiveness measures (adherence, depression severity and symptoms, health status, and health care utilization) will be analyzed similarly using linear mixed models, given the continuous nature of the measures. These models will be used to compare the average change in outcomes between study group by including time, study group, and time by study group interaction as the primary predictors of interest for each outcome. For all models, time will be measured both as survey time and time since test results were released to physicians. In the latter, data from the baseline to 6 months period for the intervention group and from 3 to 9 months for the control group will be assessed. All models will be adjusted for patient demographics.

**Table 2:** Patient participant measures of feasibility, acceptability, and preliminary effectiveness of pharmacogenomic testing.

Construct		Measure	Assessment time			
			Baseline (through paper survey)	3 months (through phone)	6 months (through phone)	9 months (control only; through phone)
**General**						
	Demographic information	Gender, age, and income	✓^a^	—^b^	—	—
**Effectiveness**						
	Depression symptoms and severity	Eight-item Personal Health Questionnaire Depression Scale	✓	✓	✓	✓
	Anxiety symptoms and severity	Generalized Anxiety Disorder 7-Item Scale	✓	✓	✓	✓
	Functional health status	12-Item Short Form Survey	✓	✓	✓	✓
	Missed days of work	Work Productivity and Activity Impairment Questionnaire	✓	✓	✓	✓
	Health history	Smoking history, body mass index	✓	—	—	—
	Health care utilization	Physician visits and hospital and emergency room admissions	✓	✓	✓	✓
	Adverse drug reactions	Antidepressant Side-Effect Checklist	✓	✓	✓	✓
	Medication and medication changes	Medications prescribed and number of medication changes	✓	✓	✓	✓
	Medication adherence	Adherence to Refills and Medication Scale	✓	✓	✓	✓
	Medication adherence	Pill count	✓	✓	✓	✓
	Medication adherence	Three-Item Self-rated Adherence Scale	✓	✓	✓	✓
**Acceptability and feasibility**						
	Pharmacogenomic knowledge, perceptions, and experiences	Investigator Developed	✓	—	✓^c^	✓

^a^✓ indicates that the construct was assessed.

^b^Not assessed at that time.

^c^Intervention arm only.

## Results

This study is ongoing. Physician enrollment occurred between November 2017 and January 2019, and we recruited 23 physician participants. Patient participant enrollment occurred between January 2018 and April 2019, and we recruited 52 patient participants. We had to withdraw 1 patient because their physician had not seen the patient clinically, nor was the physician the prescriber (see Figure 1). Recruitment for this study is now complete, and we expect patient and physician participant data collection to conclude in January 2020.

At the outset of this study, we sought to recruit patient participants who had recent diagnoses of depression and/or anxiety recorded in their medical record. However, the initial pool of potentially eligible participants was quite small, and enrollment rates were poor. In May 2018, the IRB approved protocol changes to widen the inclusion criteria to any patient with a new prescription for an antidepressant medication from one of the enrolled physicians, regardless of clinical diagnosis, as reflected in this revised protocol. This expansion in inclusion criteria resulted in some participants not having mental health–related diagnoses; however, it is likely that many participants may have undocumented depression or anxiety.

## Discussion

### Principal Findings

This paper outlines the protocol of a pilot study, which includes a wait-list randomized controlled trial investigating PGx testing for antidepressant medications in primary care. Despite commercial availability of PGx testing, institutions have been slow to adopt these tests [10]. A recent survey of 10,303 US physicians, of which 39.2% were in primary practice, found that 12.9% of respondents had ordered or recommended a PGx test within the past 6 months, and about 26.4% thought they would in the near future [21].

Despite underutilization, previous work has suggested that PGx test results could improve patient care by reducing the number of side effects, failed medication trials, and time to achieve treatment response [9]. In addition, a recent prospective 12-week study of adult patients with depression and/or anxiety treated in a variety of outpatient clinical settings reported significantly improved clinical outcomes with PGx testing. Bradley et al enrolled 685 adult patients who were randomized to have providers receive PGx test results within 1 to 3 weeks (intervention group) or not receive results (standard of care) [22]. PGx testing included pharmacokinetic and pharmacodynamic variants associated with 10 genes. Patients were either new to treatment or inadequately controlled with antidepressant medications. Patients in the intervention group had response rates (change in depression and anxiety scale scores) that were significantly higher than the standard of care group (odds ratio [OR] 4.72, 95% CI 1.93-11.52; *P*<.001 for depression and OR 1.76, 95% CI 1.03-2.99; *P*=.04 for anxiety). The proportion of patients with at least 1 medication change by week 2 was significantly higher in the intervention group than in the standard of care group (81% vs 64% at 2 weeks, respectively; *P*<.001). However, no difference in the rate of adverse reactions was reported between the study groups. Results from the study by Bradley et al support the use of PGx testing in a diverse adult outpatient population with depression and/or anxiety; however, subanalyses were not reported. Therefore, it is unclear if newly diagnosed patients (with depression and/or anxiety) or those in a primary care setting also significantly benefited from PGx testing. Moreover, this study focused only on patients with depression and/or anxiety, as opposed to all patients taking antidepressant medications regardless of diagnosis.

One of the perceived barriers to utilization of PGx testing in primary care is that clinical utility of the test has not been well established [23]. This is supported by a recent safety statement made by the Food and Drug Administration (FDA), which was made about a year after our study launched. According to the FDA, there is not sufficient evidence to know whether PGx tests influence the effectiveness or safety of antidepressant medication therapy [24]. The FDA recommends that initiation of a medication or medication changes should not be made based on PGx test results because these actions are currently not supported by sufficient scientific or clinical evidence. This highlights the pressing need for more studies to investigate the clinical utility of such tests. Data from this study will provide additional evidence to help address these key points. This trial seeks to contribute to current scientific evidence through assessing the feasibility, acceptability, and preliminary effectiveness of using PGx testing in primary care in persons newly prescribed a target antidepressant.

### Limitations

This is a pilot study with a small sample size of patient participants, which limits sample genetic variability (there are more than 50 CYP450 enzymes), the number of abnormal metabolizers, and differences in participant demographics. As previously described, we focused on CYP2C19 and CYP2D6, which metabolize the majority of the most commonly prescribed FDA-approved antidepressant medications and have CPIC guidelines to support therapeutic recommendations. In addition, because this is a pilot study, we are also limited by our relatively small sample size of patient participants who were recruited from a convenience sample of DFM physicians within our institution. Future work is needed with larger sample sizes adequate to detect a difference between groups, ensure demographic saturation, and provide a broader representation of genetic variability.

To increase enrollment, we expanded our protocol inclusion criteria from a diagnosis of depression and/or anxiety to anyone taking targeted antidepressants. The broadening of possible diagnoses increases variability in comorbidities and may change certain patient outcomes. Future studies should limit the diagnoses to avoid these variations.

As with any PGx test, there are limitations to the assay. Direct DNA testing will not detect all variants that result in decreased or increased enzyme activity. Absence of a detectable gene mutation or polymorphism does not rule out that the patient has ultrarapid, intermediate, or poor metabolizer; reduced activity; or reduced response phenotypes for these genes. More research is needed to identify all genetic variations and their possible phenotypes.

This study is being conducted in primary care clinics from 1 academic health center in the United States, which limits the generalizability of the results. Only physicians who were willing to use PGx testing for the selection of antidepressant medications were enrolled, which may be a potential bias, and limits assessment of feasibility and physician willingness to use PGx testing in clinical practice. Ideally, we would have enrolled patients before starting the antidepressant; however, delaying antidepressant therapy while waiting for PGx test results would be unethical.

At our institution, clinical pharmacists have full access to the EMR, are able to write notes, and contact prescribers using direct messaging through the EMR. These interactions are recorded within the EMR. DFM clinics are a Patient-Centered Medical Home and have clinical pharmacists practicing under a collaborative practice agreement within each clinic. Physicians are accustomed to receiving and sending information through the EMR to clinical pharmacists to facilitate patient care. Although not unique to our clinics, this practice model is not commonly used throughout the United States.

### Conclusions

This pilot study is designed to assess the feasibility, acceptability, and preliminary effectiveness of using PGx testing in a primary care setting among persons newly prescribed an antidepressant. Physicians’ and patients’ perceptions of PGx testing, as well as changes in patient medication use and outcomes, will be evaluated. Results from this study may yield positive effects for all patients on antidepressant medications, not just those with anxiety and/or depression.

## Appendix

Multimedia Appendix 1 Pharmacogenomic subjective, objective, assessment, and plan note template.

## Abbreviations

ARMS: Adherence to Refills and Medication Scale
CPIC: Clinical Pharmacogenetics Implementation Consortium
DFM: Department of Family Medicine
EMR: electronic medical record
FDA: Food and Drug Administration
GAD-7: Generalized Anxiety Disorder 7-item scale
OR: odds ratio
PGx: pharmacogenomic
PHQ-8: Patient Health Questionnaire 8-item depression scale
SOAP: Subjective, Objective, Assessment, and Plan
UM: University of Michigan

## References

[1] (2016). Centers for Disease Control and Prevention.

[2] Hirschfeld RM (2001). The comorbidity of major depression and anxiety disorders: recognition and management in primary care. Prim Care Companion J Clin Psychiatry.

[3] Mark TL, Levit KR, Buck JA (2009). Datapoints: psychotropic drug prescriptions by medical specialty. Psychiatr Serv.

[4] (2013). SAMHSA - Substance Abuse and Mental Health Services Administration.

[5] Cameron C, Habert J, Anand L, Furtado M (2014). Optimizing the management of depression: primary care experience. Psychiatry Res.

[6] Papakostas GI, Perlis RH, Scalia MJ, Petersen TJ, Fava M (2006). A meta-analysis of early sustained response rates between antidepressants and placebo for the treatment of major depressive disorder. J Clin Psychopharmacol.

[7] Posternak MA, Zimmerman M (2005). Is there a delay in the antidepressant effect? A meta-analysis. J Clin Psychiatry.

[8] Bousman CA, Forbes M, Jayaram M, Eyre H, Reynolds CF, Berk M, Hopwood M, Ng C (2017). Antidepressant prescribing in the precision medicine era: a prescriber's primer on pharmacogenetic tools. BMC Psychiatry.

[9] Milani L, Leitsalu L, Metspalu A (2015). An epidemiological perspective of personalized medicine: the Estonian experience. J Intern Med.

[10] Dunnenberger HM, Crews KR, Hoffman JM, Caudle KE, Broeckel U, Howard SC, Hunkler RJ, Klein TE, Evans WE, Relling MV (2015). Preemptive clinical pharmacogenetics implementation: current programs in five US medical centers. Annu Rev Pharmacol Toxicol.

[11] Klein ME, Parvez MM, Shin JG (2017). Clinical implementation of pharmacogenomics for personalized precision medicine: barriers and solutions. J Pharm Sci.

[12] St Sauver JL, Bielinski SJ, Olson JE, Bell EJ, Mc Gree ME, Jacobson DJ, McCormick JB, Caraballo PJ, Takahashi PY, Roger VL, Vitek CR (2016). Integrating pharmacogenomics into clinical practice: promise vs reality. Am J Med.

[13] Mikat-Stevens NA, Larson IA, Tarini BA (2015). Primary-care providers' perceived barriers to integration of genetics services: a systematic review of the literature. Genet Med.

[14] Kroenke K, Strine TW, Spitzer RL, Williams JB, Berry JT, Mokdad AH (2009). The PHQ-8 as a measure of current depression in the general population. J Affect Disord.

[15] Spitzer RL, Kroenke K, Williams JB, Löwe B (2006). A brief measure for assessing generalized anxiety disorder: the GAD-7. Arch Intern Med.

[16] Kripalani S, Risser J, Gatti ME, Jacobson TA (2009). Development and evaluation of the adherence to refills and medications scale (ARMS) among low-literacy patients with chronic disease. Value Health.

[17] (2019). CPIC: Clinical Pharmacogenetics Implementation Consortium.

[18] Huo T, Guo Y, Shenkman E, Muller K (2018). Assessing the reliability of the short form 12 (SF-12) health survey in adults with mental health conditions: a report from the wellness incentive and navigation (WIN) study. Health Qual Life Outcomes.

[19] Reilly MC, Zbrozek AS, Dukes EM (1993). The validity and reproducibility of a work productivity and activity impairment instrument. Pharmacoeconomics.

[20] Wilson IB, Lee Y, Michaud J, Fowler Jr FJ, Rogers WH (2016). Validation of a new three-item self-report measure for medication adherence. AIDS Behav.

[21] Stanek EJ, Sanders CL, Taber KA, Khalid M, Patel A, Verbrugge RR, Agatep BC, Aubert RE, Epstein RS, Frueh FW (2012). Adoption of pharmacogenomic testing by US physicians: results of a nationwide survey. Clin Pharmacol Ther.

[22] Bradley P, Shiekh M, Mehra V, Vrbicky K, Layle S, Olson MC, Maciel A, Cullors A, Garces JA, Lukowiak AA (2018). Improved efficacy with targeted pharmacogenetic-guided treatment of patients with depression and anxiety: a randomized clinical trial demonstrating clinical utility. J Psychiatr Res.

[23] Bartlett G, Antoun J, Zgheib NK (2012). Theranostics in primary care: pharmacogenomics tests and beyond. Expert Rev Mol Diagn.

[24] (2018). US Food and Drug Administration.

