# Supplementary material for: Using Pharmacogenomic Testing in Primary Care: Protocol for a Pilot Randomized Controlled Study
Source: JMIR Res Protoc. 2019 Aug 19;8(8):e13848. doi: 10.2196/13848 (PMC6764327; doi:10.2196/13848)
Supplement: Multimedia Appendix 1 [file resprot_v8i8e13848_app1.pdf]

Multimedia Appendix 1. PGx SOAP note template.

Encounter Date: @ED@

@NAME@ has met study inclusion criteria and is enrolled in the research study "Determining the Feasibility, Acceptability, and Preliminary Effectiveness of Pharmacogenomic (PGx) Testing in Primary Care."

S/O:
@NAME@ is taking *** (antidepressant) which is metabolized by ***(CYP1A2, CYP2B6, CYP2C9, CYP2C19, CYP2D6, CYP3A4). Per study protocol, ***(he/she) had a PGx test performed on ***. Patient's Department of Family Medicine Physician is ***.

Results of the PGx test indicate that this patient is a ***(Normal metabolizer, Rapid metabolizer, Intermediate metabolizer, Poor metabolizer, Unknown metabolizer) of ***(CYP1A2, CYP2B6, CYP2C9, CYP2C19, CYP2D6)

++

A/P:
The PGx test results are summarized in this table and include an interpretation based on the antidepressant medication and the patient's phenotype.

Genotype	Phenotype	Interpretation
***(*X/*X)	***(Normal metabolizer, Rapid metabolizer, Intermediate metabolizer, Poor metabolizer, Unknown metabolizer) of ***(CYP1A2, CYP2B6, CYP2C9, CYP2C19, CYP2D6)	Being a ***(Normal metabolizer, Rapid metabolizer, Intermediate metabolizer, Poor metabolizer, Unknown metabolizer) of ***(CYP1A2, CYP2B6, CYP2C9, CYP2C19, CYP2D6) is associated with ***(reduced, increased, similar) enzyme activity resulting in ***(reduced, increased, similar) blood levels of ***(antidepressant) compared with those who are normal metabolizers of ***(CYP1A2, CYP2B6, CYP2C9, CYP2C19, CYP2D6).<br><br>--***(Patients who are ***(Normal metabolizer, Rapid metabolizer, Intermediate metabolizer, Poor metabolizer, Unknown metabolizer) of ***(CYP1A2, CYP2B6, CYP2C9, CYP2C19, CYP2D6) usually require standard doses of ***(antidepressant) to achieve therapeutic concentrations. Therefore, no change is recommended.

		- Patients who are ***(Normal metabolizer, Rapid metabolizer, Intermediate metabolizer, Poor metabolizer, Unknown metabolizer) of ***(CYP1A2, CYP2B6, CYP2C9, CYP2C19, CYP2D6) require increased doses to achieve therapeutic concentrations. Therefore, the dose should be ***[insert dose here])<br><br>-***(Patients who are ***(Normal metabolizer, Rapid metabolizer, Intermediate metabolizer, Poor metabolizer, Unknown metabolizer) of ***(CYP1A2, CYP2B6, CYP2C9, CYP2C19, CYP2D6) may need a lower dosage of antidepressant to prevent adverse effects. Therefore, the dosage should be ***[insert here])<br><br>-***(Patients who are ***(Normal metabolizer, Rapid metabolizer, Intermediate metabolizer, Poor metabolizer, Unknown metabolizer) of ***(CYP1A2, CYP2B6, CYP2C9, CYP2C19, CYP2D6) may lack the ability to effectively metabolize some medications to their active component and therefore an alternative antidepressant ***[insert name here] should be used.

Based on these factors, it is recommended to:
-***According to the Clinical Pharmacogenetics Implementation Consortium (CPIC) guidelines, (Continue current therapy; Discontinue current therapy and replace with ***[insert here]); Decrease dose to; Increase dose to)***

-***Discussed results with *** [enrolled DFM physician] by phone on ***
++

The information provided here is for research purposes.
